# Using Mobile Health Tools to Engage Rural Underserved Individuals in a Diabetes Education Program in South Texas: Feasibility Study

**DOI:** 10.2196/16683

**Published:** 2020-03-24

**Authors:** Zenong Yin, Janna Lesser, Kristi A Paiva, Jose Zapata Jr, Andrea Moreno-Vasquez, Timothy J Grigsby, Stacy R Ryan-Pettes, Deborah Parra-Medina, Vanessa Estrada, Shiyu Li, Jing Wang

**Affiliations:** 1 Department of Public Health College of Health, Community and Policy University of Texas at San Antonio San Antonio, TX United States; 2 School of Nursing The University of Texas Health Science Center at San Antonio San Antonio, TX United States; 3 Department of Psychology and Neuroscience Baylor University Waco, TX United States; 4 Department of Mexican American and Latina/o Studies The University of Texas at Austin Austin, TX United States

**Keywords:** Screening, Brief Intervention, and Referral to Treatment (SBIRT), Hispanic Americans, behavioral economics, rural population, diabetes, screening

## Abstract

**Background:**

Access to diabetes education and resources for diabetes self-management is limited in rural communities, despite higher rates of diabetes in rural populations compared with urban populations. Technology and mobile health (mHealth) interventions can reduce barriers and improve access to diabetes education in rural communities. Screening, Brief Intervention, and Referral to Treatment (SBIRT) and financial incentives can be used with mHealth interventions to increase the uptake of diabetes education; however, studies have not examined their combined use for diabetes self-management in rural settings.

**Objective:**

This two-phase Stage 1 feasibility study aimed to use a mixed methods design to examine the feasibility and acceptability of an mHealth diabetes education program combining SBIRT and financial incentives to engage rural individuals.

**Methods:**

In Phase 1, we aimed to develop, adapt, and refine the intervention protocol. In Phase 2, a 3-month quasi-experimental study was conducted with individuals from 2 rural communities in South Texas. Study participants were individuals who attended free diabetes screening events in their community. Those with low or medium risk received health education material, whereas those with high risk or those with a previous diagnosis of diabetes participated in motivational interviewing and enrolled in the 6-week mHealth Diabetes Self-Management Education Program under either an unconditional or aversion incentive contract. The participants returned for a 3-month follow-up. Feasibility and acceptability of the intervention were determined by the rate of participant recruitment and retention, the fidelity of program delivery and compliance, and the participant’s satisfaction with the intervention program.

**Results:**

Of the 98 screened rural community members in South Texas, 72 individuals met the study eligibility and 62 individuals agreed to enroll in the study. The sample was predominately female and Hispanic, with an average age of 52.6 years. The feedback from study participants indicated high levels of satisfaction with the mHealth diabetes education program. In the poststudy survey, the participants reported high levels of confidence to continue lifestyle modifications, that is, weight loss, physical activity, and diet. The retention rate was 50% at the 3-month follow-up. Participation in the intervention was high at the beginning and dissipated in the later weeks regardless of the incentive contract type. Positive changes were observed in weight (mean -2.64, SD 6.01; *P*<.05) and glycemic control index (-.30; *P*<.05) in all participants from baseline to follow-up.

**Conclusions:**

The finding showed strong feasibility and acceptability of study recruitment and enrollment. The participants’ participation and retention were reasonable given the unforeseen events that impacted the study communities during the study period. Combining mHealth with SBIRT has the potential to reach individuals with need to participate in diabetes education in rural communities.

## Introduction

### Background

Obesity and type 2 diabetes mellitus (T2DM) are becoming the most prevalent chronic illnesses in the United States and worldwide [1,2]. In the United States, over 30 million people, 9.4% of the US adult population live with diabetes and 23.8% of them are undiagnosed [3]. In Texas, 11.2% of the adult population has been diagnosed with diabetes [4]. Furthermore, the prevalence of diabetes is 17% higher in rural communities than urban communities [5], and it has been repeatedly recognized as the number 3 rural health priority [6,7]. Screening is essential to identify undiagnosed diabetes, allowing individuals to access resources to manage their illness. Screening, Brief Intervention, and Referral to Treatment (SBIRT) is an evidence-based practice that involves screening patients using a validated, standardized tool, referral for patients who need additional services to brief intervention with a health care professional, and referral to treatment [8]. Traditionally used to reduce alcohol and illicit drug use, SBIRT and its benefits can be realized beyond substance abuse, and these have been applied to undiagnosed hypertension [9] and childhood obesity [10-12]. For example, Byrne et al [12] demonstrated that an electronic health screening, a brief intervention, and referral to treatment within a primary care setting were effective to engage parents of overweight children in taking preventive actions. Financial incentives, based on the principles of behavioral economics, is another strategy that has demonstrated positive effects on the uptake of lifestyle modification and disease self-management [13]. Therefore, financial incentives may play a key role in engaging low-income and minority patients in changing habitual physical activity and dietary behaviors and diabetes management skills [14,15]. To the best of our knowledge, there are no published studies that examine the use of SBIRT and financial incentives to engage rural populations in diabetes self-management.

Research has indicated that behavioral healthy lifestyle interventions that focus on self-monitoring and goal setting are effective for glycemic control [16,17]. The landmark Look AHEAD (Action for Health in Diabetes) trial demonstrated that a lifestyle intervention could achieve clinically significant weight loss and glucose control in overweight and obese adults with T2DM [18]. Previous research has documented meaningful changes in glycemic control and other diabetes-related outcomes among rural patients who participated in diabetes self-management programs offered by their primary care providers in underserved rural communities [19,20]. However, novel approaches are still needed to increase the uptake of lifestyle diabetes prevention and management programs in underserved minority and rural populations [21-24]. A recent pilot study targeting underserved individuals demonstrated the positive effects (eg, weight loss) of using mobile versus paper-based logs for diabetes self-management, indicating that technology can be integrated into interventions to assist with self-monitoring [25]. Individuals can learn how to manage their disease through diabetes education provided by a trained leader. Diabetes education is also a cost-effective way to deliver training in self-management behaviors to individuals with T2DM [26]. Although diabetes education can have a significant impact on these individuals, nearly half of the adults with diabetes in the United States have not received formal diabetes education [27].

Resources for diabetes self-management are lacking in rural communities, where the availability of diabetes education programs is sparse. People from rural areas comprise 18% of the total US population, but rural areas account for 84% of the total area in the United States [28]. Although rural populations comprise a much smaller portion of the US population, as noted above, the prevalence rate for diabetes is 17% higher in rural areas compared with nonrural areas [29]. Furthermore, although there is a higher rate of diabetes in rural populations, there are fewer medical services available, and those services are more difficult to access. In particular, southern United States is the least likely to offer diabetes education [26]. Several studies have described the disparities that exist between rural and urban regions, with rural individuals living in medically underserved areas having older individuals, fewer employment opportunities, higher rates of underinsured and uninsured, lesser educational attainment, and being more likely to live in poverty [26,29,30]. Furthermore, even when rural individuals have access to diabetes self-management programs, participation may be limited because of the distance and transportation requirements to attend the group in person [31]. One solution to improve access to diabetes self-management programs in rural areas is to use technology to deliver education. Several studies have indicated the effectiveness of technology and mHealth to reduce barriers to diabetes self-management and improve health outcomes [25,31,32].

### Study Objectives

This study combined mHealth, SBIRT, and the principles of behavioral economics to reach adults living in rural areas who were at risk for, or living with, diabetes. We tested the feasibility and acceptability of (1) recruiting rural residents to participate in diabetes screening, (2) motivating rural residents at risk for diabetes or with diabetes to make lifestyle modifications, and (3) engaging rural residents and encouraging them to complete an mHealth diabetes education program using different incentive plans. The long-term goal of the study is to develop an effective community-based diabetes education program for resource-poor rural communities at increased risk for diabetes-related health disparities. Findings from this study and lessons learned will guide future health intervention research with rural populations.

## Methods

### Study Design

This was a two-phased Stage 1 feasibility study according to the National Institutes of Health’s staged model for the development of psychosocial and behavioral interventions [33]. Using a mixed methods design, we collected both qualitative and quantitative data to evaluate the aims of the study. The study team used Phase 1 of the study (Stage IA) to identify community partners, develop recruitment plans, and adapt an integrated diabetes screening and brief intervention and motivational interviewing to a diabetes education program, with input from the community stakeholders. During Phase 2 of the study (Stage IB), we assessed the feasibility and acceptability of the diabetes education program using a quasi-experimental pretest and posttest design. The aims of Phase 2 were to gather data on the rates of study enrollment, recruitment and retention, delivery of mHealth education content, levels of engagement with the scheduled activities under either unconditional or aversion incentive contract, acceptability of and satisfaction with the diabetes screening and education program, and the sensitivity of outcomes (weight and glycemic control index) to the mHealth intervention.

### Phase 1 of the Study: Program Development and Adaptation for Delivery

During Phase 1 of the study, the study team conducted numerous site visits and successfully identified 2 community hospitals and a faith-based community nursing program as partners for conducting free diabetes screening and recruiting study participants. There was no ongoing program that offered free diabetes screening or diabetes prevention or management programs in these communities. On the basis of the information gathered from the 2 study communities, the study team developed the pilot version of the diabetes screening and education program by adapting SBIRT, financial incentives, and the mHealth diabetes education program from a previous project. We offered the pilot version of the program to 6 residents recruited from a local community. The participants completed the diabetes screening and received brief diabetes education based on their screening results. The study staff also explained the content of an mHealth education program to the participants. At the end, the study team conducted a focus group discussion with the participants to gather their feedback on the recruitment, procedure of diabetes screening and brief education, and content and delivery of the mHealth education program. The study team refined and finalized the intervention and data collection protocol based on the finding from the focus group discussion.

### Participant Recruitment in Phase 2 of the Study

Study participants lived in 2 rural communities in South Texas (Community A and Community B). Diabetes screening events were promoted by distributing recruitment flyers in community hospitals, in supermarkets, in churches, and at community events, and announcements were made on local radio stations and newspapers. To qualify for the study, participants needed to meet the following criteria: (1) live in a study community, (2) be 35 years or older, (3) own a smartphone (with a data plan) or a mobile phone (with an SMS text message plan) and have internet access via a computer or tablet, (4) complete a diabetes screening, and (5) be at high risk for diabetes, scoring >5 on the American Diabetes Association (ADA) diabetes risk test or A_1c_ level ≥5.8, or have health care provider–diagnosed diabetes. English language proficiency was not an inclusion criterion. Residents who did not meet the eligibility criteria only received diabetes screening and brief education and were not eligible to receive the study incentive. Residents who met the eligibility criteria but had low or moderate risk for diabetes or were not interested in the study received brief health counseling and health promotion materials. All eligible participants who completed diabetes screening received a US $25 grocery store gift card, and participants who completed the mHealth diabetes education program received up to US $60 in grocery store gift cards.

### Description of the Intervention: Diabetes Screening and Mobile Health Diabetes Education Program

**Figure 1:**
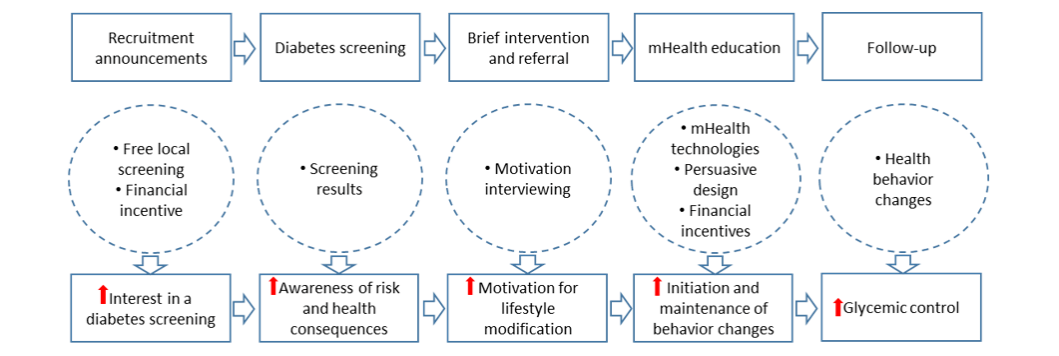
Conceptual Framework of the Diabetes Screening and mHealth Education Program.

The intervention was based on the principles of SBIRT and behavioral economics for program participants who were ready to commit to lifestyle modification with minimal social and technical support. The principles of persuasive design were used to guide the development of the mHealth diabetes education program that stresses ease of access and motivation [34]. To shed light on the intervention approach, Figure 1 shows the conceptual framework that depicts the underlying processes to engage the study participants. We conducted the Diabetes Screening and mHealth Education Program following SBIRT [8] in 3 consecutive steps, which are described in the following sections.

#### Step 1: Free Diabetes Screening

The screening was conducted by trained research assistants either in the 38-foot Mobile Health Laboratory or a large indoor space within the community. The diabetes screening included weight assessment and calculation of BMI, resting blood pressure, blood assay by finger stick for hemoglobin A_1c_, high-density lipoprotein cholesterol, total cholesterol, and glucose. Each screened participant was assessed for the risk of type 2 diabetes based on the multiple risk factor diabetes screening and education guidelines of the ADA and the US Preventive Services Task Force. Low risk was defined as scoring <5 on the ADA diabetes risk test [35], moderate risk was defined as scoring 5 on the ADA diabetes risk test, and high risk was defined as scoring >5 on the ADA diabetes risk test or an A_1c_ level ≥5.8. Participants with diabetes self-reported diagnosis of diabetes by a physician. The results of the screening were recorded on a screening report card. All participants who agreed to participate in the study were invited to complete a health needs survey and received a study information sheet. The institutional review board reviewed and approved all study protocols.

#### Step 2: Brief Intervention and Referral to Treatment

The screened residents were divided into 4 groups based on the results of the diabetes screening and willingness to participate in the study (Table 1). The study participants in groups 1 and 2 received brief education by trained research staff members (a certified community health worker or registered dietitian) who provided an explanation of screening results and brief counseling on healthy lifestyle behaviors. Each participant was provided health education brochures on various topics related to general healthy lifestyle behaviors and diabetes prevention.

**Table 1:** Grouping of study participants and participant treatment.

Screening result	Brief intervention	Referral to treatment	Incentives
Group 1: Not meeting eligibility or not interested in the study	Brief education by a community health worker (5-10 min)	No referral	US $25 grocery card for participating in screening
Group 2: low-to-medium risk for diabetes	Brief education by a community health worker (5-10 min)	No referral	US $25 grocery card for participation in screening
Group 3: high risk for diabetes	Motivational interviewing by trained research staff (15-30 min)	Mobile health Diabetes Education Program	US $25 grocery card for participation in screening Unconditional incentives (US $60) for completing a diabetes education program
Group 4: previously diagnosed diabetes	Motivational interviewing by trained research staff (15-30 min)	Mobile Health Diabetes Education Program	US $25 grocery card for participation in screening Aversion incentives (up to US $60) for completing a diabetes education program

The participants in groups 3 and 4 received brief motivational interviewing based on the Feedback, Responsibility, Advice, Menu Options, Empathy and Self-Efficacy approach that includes *Feedback* regarding demographic and biological risk for diabetes, emphasis on personal *Responsibility* and choice, *Advice* to change (when appropriate), a *Menu* of change options, an *Empathetic* listening approach, and an emphasis on *Self*-*efficacy* and optimism around change [36]. Research staff (faculty researchers, registered nursing students, and a registered dietitian) completed a 5-hour motivational interviewing training session provided by a clinical psychologist with expertise in SBIRT and motivational interviewing (MI). Trained research staff used a personalized screen report to guide the delivery of the brief MI intervention content. Section 1 of the screen report provided a health risk profile and associated health consequences based on the screening results [37]. Section 2 demonstrated the risk reduction and improvement of glycemic control based on the research evidence of lifestyle modifications (weight loss, diet, and physical activity). Section 3 included a menu of options for behavior changes and evidence-based strategies [38]. With facilitation from the research staff, participants identified behaviors deemed important to them for modification and selected goals and strategies of lifestyle change, which they were willing to attempt to adopt for the future. At the end of the brief motivation interviewing session, participants were asked to indicate their level of readiness on a scale of 0 to 10 for making lifestyle changes at the present time.

The participants with a readiness score of 5 or higher were asked if they were willing to complete a 6-week mHealth diabetes education program. Once the participants agreed to enroll in the program, a research staff member explained the details of the mHealth education program and the weekly schedules of activities. Thereafter, the study participants signed a study contract to indicate their willingness to participate in the study and complete the study activities. The content of the study contract included the following: (1) commitment to follow the schedule of program activities and (2) agreement to receive a monetary incentive for following the schedule of program activities. All participants in community A received a US $60 grocery store card after they signed the contract (unconditional incentive group). All participants in community B were promised a grocery store card worth US $60 if they completed all scheduled activities, reductions would be made for each scheduled activity that was not completed (aversion incentive group). Finally, the study participants received an information packet, which included the mHealth diabetes education program schedule, the study incentive tracking form (for community B), and study staff contact information.

#### Step 3: Delivery of the Mobile Health Diabetes Education Program

The program was delivered over a 6-week period with a different topic area for each week (see Table 2). Weekly interactive lessons were created for the participants to develop lifestyle modification knowledge and skills based on the National Diabetes Education Program and best practices for diabetes self-care [39,40]. We used Articulate Storyline (Articulate Global Inc) to develop interactive education lessons, with avatars, problem-solving quizzes, skill-building games, and embedded video clips to motivate and engage participants. At the end of each lesson, participants watched a multi-part video drama of a family dealing with diabetes. Each week, the participants were asked to complete one physical activity challenge and one diet challenge out of the 2 to 3 challenges offered, which were related to the skills presented in the lessons. Resources relevant to the topic of the week were provided to participants to further their understanding of the topic. These included YouTube videos and websites with information on physical activity, nutrition, and stress reduction. The participants accessed the content via their smartphone or internet-connected computers or tablets. Automated SMS text messages were used to send program reminders and health tips relevant to the topic of the week. The SMS text messages were delivered to participants using a reconfigurable SMS text messaging system, MessageSpace that used the “grouping” logic to define various cohorts and schedule the delivery of SMS text messages for a comparative analysis or send group-specific broadcast messaging, “polling” for collecting responses from participants, and advanced message history tracking and scheduling to determine dose/exposure.

**Table 2:** Mobile Health Diabetes Education Program curriculum.

Diabetes education lesson (to be completed by Wednesday)	Health challenge (to be completed by Sunday)	SMS text messages/polling (review or respond upon receiving)	Resources (review by Sunday)
Week 1: Understanding diabetes and obesity; eating healthy to manage or prevent obesity and diabetes	1 physical activity challenge and 1 diet challenge	2 program reminders and 2 daily texts	Videos on physical activity; 1 video on diabetes risk and consequences; and 1 website with information on diabetes risk and health consequences
Week 2: Understand what foods go in a healthy lifestyle; understand portion control and moderation	1 physical activity challenge and 1 diet challenge	2 program reminders and 2 daily texts	Videos on physical activity; 1 video on healthy eating; and 1 website with information on healthy eating strategies
Week 3: Learning important nutrition terms; learn how to read a nutrition label; and learn what is healthy vs unhealthy	1 physical activity challenge and 1 diet challenge	2 program reminders and 2 daily texts	Videos on physical activity and 1 website with information on nutrition facts
Week 4: Learn what counts as physical activity; learn how physical activity helps diabetes; choose an activity that is fun for you; and class stretching activity	1 physical activity challenge and 1 diet challenge	2 program reminders and 2 daily texts	Videos on physical activity; 1 website with information on physical activity
Week 5: Benefits of stress reduction; recognize symptoms of depression; how to reduce stress; importance of socializing; how to meet new people; and benefits of enough sleep	1 physical activity challenge and 1 diet challenge	2 program reminders and 2 daily texts	Videos on physical activity; 1 video on depression; 1 website with information on stress management; 1 breath control exercise video
Week 6: Diabetes myths; create a healthy plate for managing diabetes; and exercise and food for managing diabetes	1 physical activity challenge and 1 diet challenge	2 program reminders and 2 daily texts	Videos on physical activity; 1 video on diabetes management; 1 website with information on diabetes self-management

At the beginning of each week (Monday), the participants received an SMS text message to notify them about the educational activities for the upcoming week and a link to a REDCap portal that provided access to all educational content. An email with the same information was also sent to the participant’s email address. The participants were asked to submit a report on the REDCap portal to the research team to acknowledge the receipt of class activities and report any technical issues. At the end of each week (Sunday), participants received an SMS text message to indicate whether they had completed the activities and encourage them to complete the activities if they had not done so. If there was no indication of participation for more than one week, a research staff member called the participants and asked whether they needed help. The participants were also encouraged to call the study team if they needed technical assistance.

After completing the 6-week program, participants received weekly SMS text messages with health tips for an additional 6 weeks. The study participants returned to participate in a follow-up screening event (ie, the posttest) at the end of the 3-month study period. They were debriefed on the study and received additional information on lifestyle modification strategies and local health resource maps to support their continued effort of lifestyle change. The participants in the aversion group also received their grocery card incentive.

### Measurement and Evaluation

#### Evaluation of the Feasibility of Study Implementation

The information used to assess the feasibility of study implementation included (1) characteristics of the residents interested in the study, (2) feasibility of recruitment strategies and participation and retention of study participants, (3) participant engagement and compliance with the intervention, (4) fidelity of intervention delivery, (5) standardization of the intervention protocol, (6) refinement of outcome measurement and process evaluation protocols, as well as staff training program, and (7) completion rate of biometric measures.

#### Evaluation of Acceptability of the Intervention

After completing the follow-up diabetes screening, the participants were invited to complete an anonymous survey to obtain their perception of the program benefits, satisfaction with program activities, and evaluate their confidence to continue efforts to maintain a healthy lifestyle, and a 15-to-20-min debriefing interview was conducted with a faculty researcher.

#### Biometric Data

All participants completed 2 diabetes screenings that included biometric information at baseline and 3-month follow-up. Data were collected following a standardized protocol by trained research assistants in the University of Texas at San Antonio Mobile Health Laboratory, in a 38-foot customized recreation vehicle, or within the space provided by the study partners in a community setting. The protocol was also piloted using both the Mobile Health Laboratory and indoor space to identify the most efficient use of space and staff. Height and weight were measured twice, and participants wore light clothing. BMI (in kg/m^2^) was calculated by taking the average of two measures, which required discrepancies of less than 0.5 kg and 0.5 cm for weight and height measurements, respectively. Hemoglobin A_1c_ (A_1c_) was assessed by using a finger-stick testing machine (A1CNow+ System, PTS Diagnostics). In a randomized controlled trial (RCT), BMI for participants at risk for diabetes and A_1c_ for participants with diabetes would be the primary outcome measures to assess the efficacy of the intervention program. Resting systolic and diastolic blood pressure was measured using an electronic blood pressure monitor after a 5-min rest. Three measures were taken; the two closest measures were averaged. We did not include the blood pressure data in the analysis as the testing environment varied from baseline to follow-up screening.

### Data Analysis

Descriptive statistics were calculated for feasibility as well as demographic and biometric data by community location (incentive groups) and for the total sample. Furthermore, *t* tests (two-tailed) for continuous variables and chi-square tests for categorical variables were used to determine the demographic and biometric differences between the incentive groups over time (baseline to follow-up). Analysis of variance was performed to test the changes in the biometric data from baseline to follow-up, controlling for significant demographic covariates (*P*>.25). Analyses were performed in SPSS version 23 (IBM Corp, 2017) and Stata version 14 (Stata Corp, 2017). Content analysis was used by a faculty researcher to analyze the participant responses in the follow-up interviews [41].

## Results

### Phase 2: Study Recruitment and Program Enrollment

**Figure 2:**
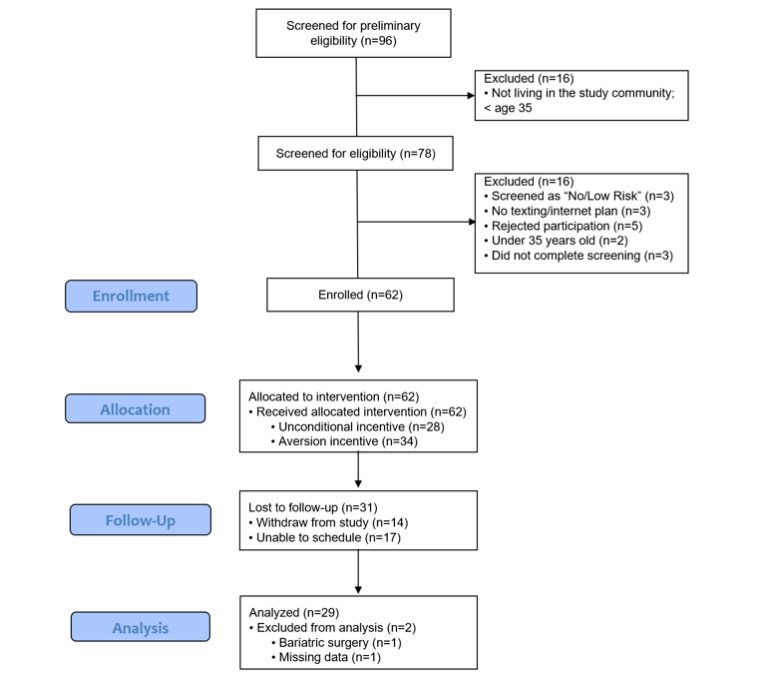
Study Participant Flow.

The diabetes screening protocol was designed to resemble diabetes screenings offered in community or worksite health fairs. The quality of the screening was enhanced by offering a brief intervention and referral to treatment based on the screening outcomes. The characteristics of the residents attending the screening events and the study sample in the 2 study communities are shown in Table 3. The screening goal was 100 residents who would attend the free screening events. We conducted 6 screening events in the 2 communities (3 in community A and 3 in community B), with 98 residents attending the events. A total of 72 of these residents met the preliminary eligibility and completed some or all of the diabetes screening. A total of 62 of the screened residents (28 in community A, the unconditional incentive group, and 34 in community B, the aversion incentive group) enrolled in the mHealth diabetes education program and became study participants (yield rate of 67.4%). There was no difference in demographic and biometric variables between screened and study participants. Over 70% (22/79) of the participants were female, primarily Hispanic, had a family history of diabetes, and accessed the internet with a smartphone. A total of 69% of the participants were 45 years old or older. Less than 6% of the participants reported speaking Spanish at home or using Spanish for reading. Over 25% of the participants did not have a primary care provider. The average A_1c_ was 6.9 (SD 3.4) and 6.5 (SD 2.0) at baseline for the screened participants and study participants, respectively. The demographic and health characteristics of the study participants in community A and community B were similar, with the exception that the rate of diagnosed diabetes (*P*<.01) and BMI (*P*<.05) were higher in the latter. Figure 2 shows the flow of the study participants.

**Table 3:** Characteristics of study sample and screened residents.

Variable^a^		Unconditional incentive (n=28)	Aversion incentive (n=34)	Total study sample (n=62)	Attendants of all events (n=78)
Female gender, n (%)		22 (79)	23 (68)	45 (73)	54 (69)
Hispanic, n (%)		21 (75)	20 (74)	46 (74)	55 (71)
Family history of diabetes, n (%)		20 (71)	29 (85)	49 (79)	58 (75)
Gestational diabetes, n (%)		3 (14)	7 (30)	10 (22)	14 (18)
Diabetes diagnosis^b^, n (%)		3 (11)	14 (41)	17 (27)	24 (31)
Participated with others, n (%)		12 (43)	15 (44)	27 (44)	27 (35)
Owning a cell phone, n (%)		27 (96)	33 (97)	60 (97)	73 (94)
Having an email, n (%)		22 (79)	24 (71)	46 (74)	53 (68)
**Language spoken at home, n (%)**					
	English	25 (89)	29 (85)	54 (87)	65 (83)
	Spanish	1 (4)	3 (9)	4 (7)	4 (5)
	Both	1 (4)	1 (3)	2 (3)	5 (6)
	Other language	0 (0)	0 (0)	0 (0)	0 (0)
**Language used for reading, n (%)**					
	English	25 (89)	29 (85)	54 (87)	65 (83)
	Spanish	1 (4)	3 (9)	4 (7)	4 (5)
	Both	1 (4)	1 (3)	2 (3)	2 (3)
	Other language	0 (0)	0 (0)	0 (0)	0 (0)
Having a primary care provider		22 (79)	24 (71)	46 (74)	55 (71)
Age (years), mean (SD)		53.9 (11)	51.5 (11)	52.6 (11)	52.7 (12)
Weight (lbs), mean (SD)		194.8 (33)	216.4 (58)	207.4 (50)	206.7 (49)
BMI (kg/m^2^)^c^, mean (SD)		30.4 (10)	36.6 (8)	34.1 (9)	34.5 (8)
Total cholesterol, mean (SD)		187.9 (64)	165.5 (34)	173.3 (47)	173.3 (47)
High-density lipoprotein, mean (SD)		52.7 (16)	46.8 (16)	48.8 (16)	48.8 (16)
Hemoglobin A_1c_, mean (SD)		6.2 (2)	7.4 (4)	6.9 (3)	6.5 (2)

^a^Chi-square test for categorical variables and independent *t* test for continuous variables for comparison of the treatment groups.

^b^*P*<.01.

^c^*P*<.05.

### Phase 2: Participant Engagement and Retention

The mHealth Diabetes Education Program was delivered to the participants in 4 cohorts starting in late July 2017. Hurricane Harvey, a Category-4 hurricane (August 17,2017-September 2, 2017) landed on the Texas coastline and caused historic flooding and interruptions in mobile phone and internet services in South Texas, including the study communities. During this period, 14 participants dropped out of the study. Although we were not certain to what extent this inopportune event impacted the participants’ participation in the mHealth Diabetes Education Program, the expectation that the study participants would adhere to the scheduled intervention activities seemed highly inappropriate. The impact was severe in the later cohorts. This prevented us from being able to evaluate the effects of the financial incentives on the participant’s engagement in the program. To gauge the levels of participation, we tracked the participants’ responses to the scheduled activities each week as indicators of intervention compliance. Table 4 shows the number of weeks (mean 2.5, SD 2.31 weeks, for the total sample) the participants responded to the intervention activities, which is measured by a record of a reported participation in at least one activity from the REDCap portal, a reply to an SMS text message, or telephone contact with the participant.

**Table 4:** Number of weeks the participants responded to the intervention activities by treatment group.

Number of weeks responded	Unconditional incentive (n=28)	Aversion incentive (n=34)	Total sample (N=62)
0, n (%)	5 (18)	10 (29)	15 (24)
≥1, n (%)	23 (82)	24 (70)	47 (76)
≥2, n (%)	19 (68)	21 (61)	40 (65)
≥3, n (%)	15 (54)	19 (55)	34 (55)
≥4, n (%)	11 (39)	17 (50)	28 (45)
≥5, n (%)	9 (32)	14 (41)	23 (37)
≥6, n (%)	6 (21)	7 (21)	13 (21)
Average of 6, mean (SD)	2.5 (2.2)	2.4 (2.3)	2.5 (2.1)

An additional analysis showed that the persons who participated in the study with relatives or friends (mean 3.2, SD 2.35) participated more often than those who participated in the study alone (mean 2.0, SD 1.92; t_60_=2.17; *P*<.03). Table 5 shows the response rate to the intervention activities, indicated by how many participants participated in the intervention by week. The average weekly response rate of 4 cohorts was 41.4% for the total sample. There were no discernible differences by cohort or between the 2 incentive groups.

The 12-week follow-up screening was planned as a celebration of program completion and evaluation of progress in lifestyle modification. In total, 31 of 62 study participants (response rate: 50%) attending the follow-up screening. During the first week of the follow-up screening, a mass shooting killed 26 adults and children and wounded 20 others in a church less than 30 miles away from the 2 study communities. We decided to terminate the follow-up screening, which seemed inappropriate given the social and psychological impact of the shooting on the community. We attributed the low retention rate partly to this shooting event as well as the occurrence of a hurricane early in the intervention phase.

**Table 5:** Response rate to the intervention activities by week by the treatment groups

Study week	Unconditional incentive (n=28), n (%)	Aversion incentive (n=34), n (%)	Total sample (N=62), n (%)
1	19 (67)	24 (71)	43 (69)
2	12 (43)	13 (38)	25 (40)
3	11 (39)	14 (41)	25 (40)
4	13 (46)	11 (32)	24 (38)
5	9 (32)	11 (32)	20 (32)
6	6 (21)	11 (32)	17 (27)
Average of 6 weeks	12 (42)	14 (41)	26 (41)

### Feasibility and Acceptance of Diabetes Screening Protocol in Phase 2 of the Study

The screening protocol was efficient to screen the number of participants, with a team of 5 trained research assistants for collecting the biometric data, 2 community health workers for delivering brief education to low-to-moderate risk residents, and 2 or 3 trained research staff for motivational interviewing with high-risk and diabetic participants. The use of the Mobile Health Laboratory offered a standardized environment to collect quality data for evaluation of the program’s impact on glycemic control and body weight. To maintain high reliability and reduce testing interferences from the surrounding environment and weather, all biometric data, especially finger-stick tests and blood pressure, were collected from the Mobile Health Laboratory.

The completion rate of weight and A_1c_ measurement was 93.5% at baseline and 93.6% at 12-week follow-up. The main reasons for missing the weight and A_1c_ were the failure to collect data from the diabetes screening report and to run the blood assay using finger-stick tests. Some blood pressure measurements were not collected. A total of 38% (24/62) of the participants at baseline did not fast overnight. The completion rate of blood pressure and lipid measure was less than 70%. As a result, blood pressure and fasting glucose were not included in the analysis. There was no adverse event during the screening events. A total of 5 of 17 (29.4%) participants with A_1c_ ≥6.5 had not been diagnosed previously as having diabetes and were referred to treatment at the 2 community hospitals.

### Fidelity of Implementation of Diabetes Screening and Mobile Health Education Program in Phase 2 of the Study

All program activities were implemented as scheduled by the research team. The REDCap portal worked effectively in presenting the program activities to the participants in a standardized format. There was no report of difficulty in navigating the content on the REDCap portal. However, the study participants in their follow-up interviews reported that weak smartphone signals and unstable internet download speed caused difficulty in completing the interactive health lessons and interrupted their viewing of the videos, and others complained of not being able to see the content in the REDCap portal because of formatting issues on their phone. Records from MessageSpace showed that out of the 85 SMS text messages delivered, 49% of the participants received all of the SMS text messages, 43% of the participants received 75% of the texts, and 8% of the participants received ≤50% of the texts. Anecdotally, the participants reported difficulty in understanding the instructions for completing the activities; therefore, they often did not submit the report on the REDCap portal even if they had reviewed the content.

### Acceptability of Diabetes Screening and Mobile Health Education Program in Phase 2 of the Study

Table 6 shows the results of the poststudy survey that participants completed at the follow-up screening. The participants reported that the program helped them to be more active than before, eat healthy, and lose weight, with 73.6% to 94.1% of the participants indicating responses from “agree” to “strongly agree.” The participants reported high levels of satisfaction with the program content (interactive lessons, SMS text messages, and videos). They indicated confidence to continue lifestyle modification with >85% indicating responses from “agree” to “strongly agree.” Finally, the participants were asked to indicate if exercising regularly, eating healthy, or losing weight is now an essential, high, moderate, low, or no priority activity compared with the beginning of the program. The majority of the participants indicated that exercising regularly (73%), eating healthy (74%), and losing weight (77%) had become essential or high priority (data not shown). Overall, the participants were least satisfied with the weight loss component of the program.

**Table 6:** Poststudy survey of the diabetes education program (n=25).

Questions		Strongly agree, n (%)	Agree, n (%)	Disagree/strongly disagree, n (%)
**Please answer these questions as honestly as possible.**				
	Did the eDiabetes Education Program help you to be more physically active?	8 (32)	14 (55)	3 (12)
	Are you still being active with the information from the eDiabetes Education Program?	10 (38)	13 (53)	2 (9)
	Did the eDiabetes Education Program help you to eat healthy?	10 (38)	14 (56)	1 (6)
	Are you still eating healthy with the information from the eDiabetes Education Program?	8 (32)	15 (59)	2 (9)
	Did the eDiabetes Education Program help you to lose weight?	6 (24)	12 (50)	7 (26)
	Are you still trying to lose weight with the information from the eDiabetes Education Program?	7 (27)	14 (56)	4 (18)
	I liked the weekly diabetes education lessons.	8 (32)	17 (68)	0 (0)
	I learned how to change my lifestyle with information from the health education lessons.	9 (35)	15 (59)	1 (6)
	I liked the weekly text messages with health tips.	12 (49)	12 (49)	1 (3)
	I liked the weekly health challenges.	9 (34)	15 (60)	1 (6)
	I liked the YouTube videos on physical activity and diet.	8 (30)	14 (58)	3 (12)
	I liked the YouTube videos with information on obesity and diabetes.	8 (30)	13 (55)	4 (15)
**Compared with when the eDiabetes Education Program started in the summer...**				
	I am confident that I can continue to exercise regularly	11 (43)	13 (51)	1 (6)
	I am confident that I can continue eating healthily	10 (40)	14 (54)	1 (6)
	I am confident that I can continue to lose weight	11 (43)	11 (43)	3 (14)

The themes generated from the follow-up interviews with 13 participants are shown in Table 7. The participants perceived that the program was motivational and had positive effects on their lifestyle modification effort. Their experience was negatively influenced by technical issues with slow internet speed, weak smartphone signals, and lack of Web-based support. They encountered various nontechnological barriers to complete the program activities, such as family commitment and work conflicts. The impact of the hurricane also prevented some from continuing the program. Respondents stated that the program could be improved by offering some human interactions with the participants, improving the interface on REDCap portal to reduce participant burden, and providing training on “how to do things.”

**Table 7:** Themes of participant interviews and exemplar statements (n=13).

Themes	Number of responses	Exemplars
Positive experiences with the program	38	“I have eaten more fruit than I ever have, watch my soda intake and eat steamed vegetables. My wife cooks differently now” “Changing my diet and walking. Tried to eat more healthy meals. Helpful to do with the family” “I did the challenges. When I first did the challenges, I picked 20 minutes. Now it is nothing. I used to have no energy and now I do”
Motivators	19	“I think if it were not for the texts message, I would have let go long ago” “I knew I wasn’t eating well, I wanted to change my diet and my husband’s” “Starting this motivated me. When I came here [for the initial screening], my weight was so high!”
Technological issues	20	“The video kept freezing. It was really frustrating” “When I got the texts, I could not see all of it. It was frustrating…I was trying so hard” “Had it on the phone. More adapted to phone. Had a lot of trouble with computer” “The videos were the least useful… basic and banal…hard to watch”
Circumstances undermining the ability to engage as anticipated	10	“The hurricane damaged my home…hard to catch up. I have custody of my grandchildren. I am always working and tired” “At the beginning I tried to do all the challenges, towards the end I wasn’t. Summer vacation was over, when I went back to work, I could not do anymore. Beginning of the school year is a stressful time” “This got put on the back burner, I was working fulltime, going to school, two children, and we were moving”
Suggestions to improve the program	11	“Have activities in town, especially for senior citizens. Face-to-face exercising with a group” “Have a more user-friendly way of showing participants what challenges they have and have not completed” “Have people keep a log, food journaling” “Improve how we submit our progress online

### Sensitivity of the Study Outcome Measures to the Diabetes Screening and Mobile Health Education Program

Table 8 displays the unadjusted changes in the study outcomes from baseline to follow-up between the incentive groups and the total sample. Overall, all of the outcome measures showed positive responsiveness to the intervention with a significant reduction in weight (*P*<.05) in the total study sample from baseline to follow-up over a 12-week period (see Table 8).

**Table 8:** Unadjusted changes of the outcome measures from baseline to follow-up by treatment group and for total study sample.

Variable^a^	Unconditional incentive		Aversion incentive		Total sample	
	n	Mean (SD)	n	Mean (SD)	N	Mean (SD)
Weight (lbs)	13	–3.88 (3.44)	16	–1.63 (7.46)	29	–2.64^a^ (6.01)
BMI (kg/m^2^)	13	–0.24 (1.53)	16	–0.03 (1.62)	29	–0.12 (1.55)
Total cholesterol	6	–42.33 (116.97)	13	–2.15 (26.44)	19	–14.84 (68.08)
High-density lipoprotein	6	4.71 (18.20)	14	3.45 (7.82)	20	3.89 (11.98)
Hemoglobin A_1c_^b^	13	0.18 (0.65)	16	–0.68 (1.29)	29	–0.30 (1.12)

^a^The analysis of variance for comparing the changes between the treatment groups; paired *t* test for comparing the changes in the total sample from the baseline to the posttest.

^b^*P*<.05.

Interferences in participants’ responses to the scheduled activities due to hurricane-related flooding and small sample size due to low retention made the comparison by the incentive group not meaningful. Therefore, the effects of the 2 incentive plans on the outcome measures including a significant reduction in A_1c_ in community B compared with that in community A should be interpreted with caution.

## Discussion

### Principal Findings

Findings from this feasibility study lend support to the conceptual framework of the intervention and the feasibility and acceptability of combining SBIRT, the components of behavioral economics, and an mHealth diabetes education in underserved rural communities. The protocol for recruitment and program enrollment was successful in generating the targeted study sample, but the retention rate can be improved through the lessons learned. Using both qualitative and quantitative data, the study demonstrated high levels of satisfaction and acceptability of free diabetes screening and brief counseling among the rural residents. In light of numerous challenges encountered in the delivery of the mHealth content, participants’ responses to intervention activities were mostly favorable. The study team gained important insights to address the barriers and improve the mHealth intervention for this underserved rural population.

The findings of the study supported the premise and conceptual framework of the intervention. The participants reported high levels of satisfaction with the program content as well as increases in their abilities and priorities to improve physical activity, diet, and weight loss as a result of the intervention. Satisfaction with the program and changes in behavioral intentions are important mediators and moderators of program effectiveness and are associated with positive behavior change outcomes [42,43].

It was important to this study to determine the feasibility of recruiting and engaging rural residents of 2 communities with largely Hispanic populations into an mHealth diabetes prevention program. The feasibility of recruitment was demonstrated by screening 96 residents within the 6 scheduled recruiting events. Of those screened, 62 enrolled in the study for an enrollment rate of 65%, which is comparable with other studies engaging rural Hispanic individuals [28-30]. However, this study had a low retention rate (50%) compared with a similar mHealth diabetes intervention conducted with a disparate population [19]. The high rate of attrition in this study may be attributed to the ramifications of Hurricane Harvey, which occurred in August 2017, and the Sutherland Springs shooting, which occurred within 30 miles of the data collection site, during the time this pilot study was conducted [31].

Although enrollment rates were sufficient for this two-phase study, future studies will require higher enrollment and retention to achieve sufficient power. Research has shown that word-of-mouth recruitment is an effective method for recruiting rural Hispanic populations, particularly Spanish-speaking first-generation immigrants, to participate in research [44]. Others have also shown that utilizing members of the community increased the rapport between recruiters and participants and should be considered as methods to increase enrollment and retention in future studies [45,46]. Furthermore, retention may be increased by using community health workers (CHWs) or promotoras. A CHW or promotora is a frontline public health worker who is a trusted member and/or has an unusually close understanding of the community served. This trusting relationship enables the CHW to serve as a link between health services and the community to facilitate access to services and improve the quality and cultural competence of service delivery [35]. A CHW also builds individual and community capacity by increasing health knowledge and self-sufficiency through a range of activities such as outreach, community education, informal counseling, social support, and advocacy [35]. Previous studies have demonstrated that retention in diabetes self-management programs is higher when promotoras are used compared with lay leaders [8] and that promotoras are trusted and provide social support in a different way than does a traditional provider [9]. As our study focused on electronic communication to engage participants, a hybrid “blended” model that utilizes CHWs to screen and deliver brief interventions, as well as provide follow-up throughout the study, has the potential to increase engagement and retention for the duration of an mHealth study [36].

The low response rate to the scheduled intervention activities cannot be fully attributed to differences in incentives in consideration of the interruptions from the hurricane-related flooding in the community. Furthermore, programs that emphasize knowledge acquisition without including behavior change have proven to be less effective in obtaining positive outcomes. There is agreement that education in itself does not lead to behavior change, as there are many factors both internal and external to an individual that lead to changes in health behaviors [10]. For example, goal setting has led to the attainment of behavior change goals for individuals with diabetes [11], and interventions focused on self-management that include both education and behavior change techniques have proved to lead to improvements in health outcomes, such as weight loss in individuals with diabetes [19]. Social support has been identified as a critical factor in mHealth interventions [19]. In this study, participants who enrolled in the program with a friend or family member had a higher response rate compared with those who participated alone. As such, to successfully change behaviors and obtain health outcomes, it will be necessary in future studies to focus on multiple factors that lead to behavior adoption and maintenance.

### Limitations

There are several limitations that should be considered when interpreting the findings of this project. First, we collected data from a convenience sample and the findings may not generalize to adult residents of rural areas in the United States. Second, we did not collect process data to examine the usability of the platform delivering the intervention content, which could provide information on the participant’s acceptability of the intervention [47] Third, study participants’ health insurance status was not collected, this information could have strengthened the study findings and better informed the targeted intervention based on insurance status in the future. Fourth, due to the exploratory nature of this project and the focus of the study on feasibility and acceptability, the statistical findings need to be interpreted with caution. Additionally, we had high attrition between the 2 time points in part due to 2 local disasters during the study period. Interpretations of study findings need to consider the impact of these 2 local disasters.

### Conclusions

This pilot study demonstrated strong feasibility in recruiting rural patients with diabetes and delivering a technology-based lifestyle intervention, despite numerous challenges. The acceptability of the program was high. The participants perceived the program was motivational and had positive effects on their lifestyle modification effort. The feasibility and acceptability information collected from this mixed methods study generated meaningful insights for planning a large scale RCT of this intervention and suggested adding a human touch, for example, through CHWs. Future studies should examine the mechanisms of the mHealth intervention that influence the outcomes in diabetes control in this underserved rural diabetes population.

## Abbreviations

ADA: American Diabetes Association
CHW: community health worker
mHealth: mobile health
MI: motivational interviewing
RCT: randomized controlled trial
SBIRT: Screening, Brief Intervention, and Referral to Treatment
T2DM: type 2 diabetes mellitus
